# Air-Dried Human Amniotic Membranes: Sterility, Microbial Barrier, and Cytokine Retention

**DOI:** 10.7759/cureus.91379

**Published:** 2025-09-01

**Authors:** Fawzi Ebrahim, Mohamed B Milad, Mouldi Saidi, Adam Elzagheid

**Affiliations:** 1 Faculty of Sciences of Tunis, University of Tunis El Manar, Tunis, TUN; 2 Cell and Tissue Culture, Libyan Biotechnology Research Center, Tripoli, LBY; 3 Science and Nuclear Technology, National Center for Science and Nuclear Technology, Tunis, TUN; 4 Genetic Engineering, Libyan Biotechnology Research Center, Tripoli, LBY

**Keywords:** air-drying, amniotic membrane grafts, cytokines, gamma radiation, growth factors

## Abstract

This study aimed to evaluate the long-term structural integrity, sterility, and bioactivity of air-dried human amniotic membranes (AD-hAM) sterilized with gamma radiation after storage for one and two years. hAM were obtained following elective cesarean delivery under sterile conditions, processed using standardized air-drying protocols, and sterilized with gamma radiation at 15, 20, or 25 kGy. Histological examination showed preservation of the stromal matrix, despite partial epithelial detachment and reduced cell density after air-drying compared with frozen hAM (F-hAM). Bioburden testing confirmed the absence of microbial contamination before and after gamma irradiation and after one year of storage at 4°C. Microbial impermeability assays further demonstrated that irradiated membranes remained effective barriers against several bacterial types even after two years of storage at 4°C. Total protein content and levels of basic fibroblast growth factor and transforming growth factor-beta declined significantly in a dose-dependent manner (p < 0.05) following air-drying and irradiation. Despite this decline, key structural features and barrier function were preserved. These findings validate air-drying combined with gamma sterilization as an effective method for producing sterile, shelf-stable hAM grafts with preserved mechanical integrity, supporting their use as safe biological dressings. However, the observed reduction in protein and cytokine content highlights the need to balance sterilization efficacy with bioactivity preservation.

## Introduction

In recent years, human amniotic membranes (hAM) have been extensively studied and applied as a biomaterial in various medical and surgical fields. The main function of hAM is to protect the fetus from external factors during intrauterine development, providing both mechanical support and biochemical signaling through its rich composition of growth factors, cytokines, and extracellular matrix components [1]. Structurally, hAM is relatively simple, consisting of an epithelial layer and an underlying stroma. The stroma is composed of the basement membrane, compact layer, fibroblast layer, and spongy layer [2]. The smooth inner surface of hAM forms a thin, tensile, avascular, semi-transparent structure devoid of nerves, muscle, and lymphatics. hAM possesses several clinically valuable properties. It is anti-inflammatory, antibacterial, antiviral, anti-fibrotic, and anti-scarring. It promotes epithelialization, reduces inflammation and fibrosis, enhances neovascularization, provides a substrate for cell growth, and functions as a biological bandage [3-6]. In addition, it contains growth factors, cytokines, and cells with stem cell-like properties [7], along with favorable physical qualities such as permeability, stability, elasticity, flexibility, and resorbability [8,9]. Its safety has been demonstrated in clinical applications [10]. Multiple studies have shown that hAM produces growth factors that promote angiogenesis, re-epithelialization, immunomodulation, cell migration, and proliferation, processes essential for wound healing [11-13]. These regenerative effects are largely mediated by growth factors such as epidermal growth factor, keratinocyte growth factor, hepatocyte growth factor, and basic fibroblast growth factor (bFGF) [14]. During wound healing, bFGF is critical for granulation tissue formation, supporting re-epithelialization and tissue remodeling, while transforming growth factor-beta (TGF-β) helps preserve extracellular matrix integrity and stimulates collagen production [15].

These properties make hAM a promising natural scaffold for tissue engineering. It has been effectively used in the treatment of burns, ulcers, bedsores, and diabetic foot ulcers [16]. However, freshly collected hAM is rarely used directly in clinical practice due to the risks of disease transmission and post-transplant complications [17]. To address this, several preservation methods have been developed, including cryopreservation, freeze-drying (lyophilization), and air-drying, all of which may affect the biological properties of hAM [18]. Air-drying is an economical method that produces a ready-to-use product suitable for room-temperature storage. Typically, hAM is dried in a laminar flow hood at room temperature for varying durations [19]. Commercially available air-dried and frozen hAM (AD-hAM and F-hAM) products are sterilized using standardized methods. Drying reduces the irritation associated with fresh samples and minimizes storage challenges, making hAM safer and more practical for medical applications [20]. However, the sterilization process must preserve the membrane’s biological activity [21,22]. Tissue allograft sterilization can be achieved through chemicals, heat, UV, gamma radiation, or electron beam (E-beam). Among these, gamma radiation is considered the most reliable and effective method [23]. Comparative studies show that gamma radiation offers several advantages over heat and chemical sterilization, as it avoids high temperatures and toxic by-products while providing strong antibacterial, antiviral, and antifungal effects at doses around 25 kGy [24-26]. The preferred radiation source is cobalt-60 (60Co) [27]. Importantly, gamma sterilization does not adversely affect the key physical or biological properties of hAM [28]. Here, we present an economical and simple processing protocol for hAM based on air-drying and gamma irradiation, and investigate its effects on growth factor levels.

The aim of this study was to establish a standardized, cost-effective, and efficient protocol for processing and storing hAM to ensure compatibility with clinical applications. Specifically, we evaluated the effects of air-drying, gamma irradiation, and prolonged storage on sterility, microbial barrier integrity, and biological activity, with a focus on key regenerative cytokines.

## Materials and methods

Harvesting and transporting placentas

The study was approved by the Bioethics Committee at the Biotechnology Research Center, Tripoli, Libya (Ref No. BEC-BTRC6-2021). The protocol complied with the World Medical Association Declaration of Helsinki (Ethical Principles for Medical Research Involving Human Subjects). Written informed consent was obtained from all participants.

Human placentas were collected from the maternity department of Ghout Shaal Clinic, Tripoli, Libya, between October 2022 and November 2023. Donors were healthy, full-term women undergoing elective cesarean section, all of whom were screened during pregnancy and/or prior to surgery for HIV, HBV, HCV, and syphilis.

Exclusion criteria included uncontrolled pregnancy, acute or chronic maternal or fetal infections, infection or inflammation of the fetal membranes, physical or mental disabilities in the mother, and maternal age below 18 years. An obstetrician confirmed adherence to the inclusion criteria and provided follow-up at the same clinic. The study enrolled 30 donors aged 25-44 years, all of whom delivered at term without a history of pregnancy complications. A total of 32 placentas were collected, of which four were excluded due to incomplete medical histories. All remaining placentas met the inclusion criteria.

Placentas were collected and transported under aseptic conditions at a controlled temperature of 4°C to the laboratory. Tissue was transported in phosphate buffer containing penicillin (10,000 IU/mL), streptomycin (10,000 µg/mL), and amphotericin B (2.5 µg/mL) (BioLife, USA) and delivered to the Cell and Tissue Culture Laboratory at the Libyan Biotechnology Research Center (LBTRC), Tripoli. Tissue processing was initiated immediately upon arrival, within 4-6 hours of delivery.

Amnion processing

**Figure 1:**
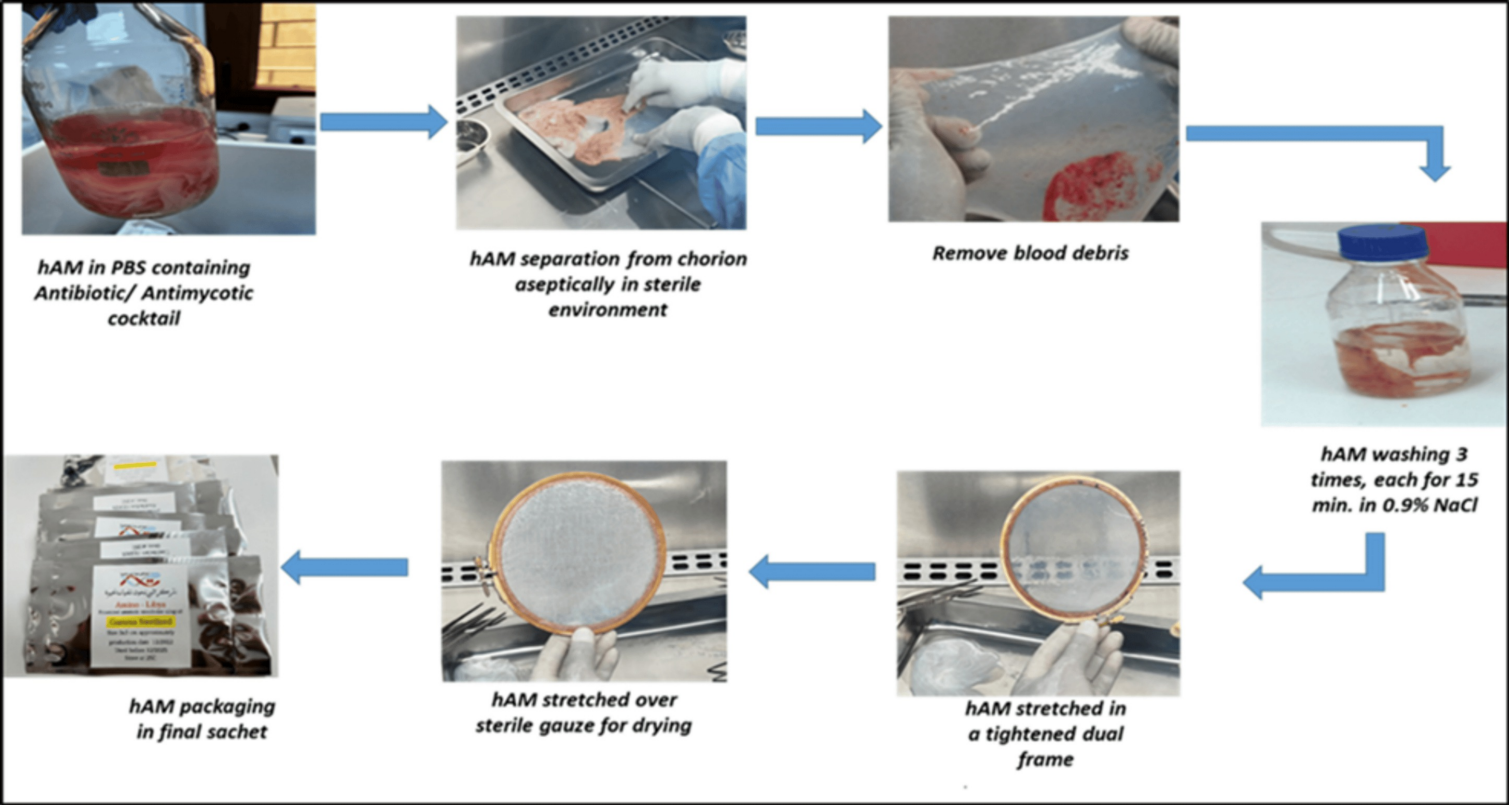
Schematic representation illustrating the steps in preparing air-dried human amniotic membranes (AD-hAM). The process includes receiving the amnion, sequential washing, air-drying, and packaging in sterile plastic envelopes. All procedures were performed under aseptic conditions in a Class II biosafety cabinet. Image Credit: Authors' original creation.

hAM was prepared according to the IAEA protocol (1997). The membrane was separated from the chorion by blunt dissection in stainless steel pans under a Class II safety hood (Telstar, Spain). Fresh placentas were washed three times with sterile saline solution (0.9% NaCl) at room temperature. The washing and shaking steps were repeated to remove blood clots and debris. Finally, the amnion was rinsed with sterile saline solution containing antibiotics (penicillin 10,000 IU/mL, streptomycin 10,000 μg/mL, and amphotericin B 2.5 µg/mL) and stored at 4°C [29] (Figure 1). All images presented in this article were captured at the Cell and Tissue Culture Laboratories of the LBTRC. Copyright is held by the Center.

Large pieces of hAM were stretched onto sterilized dual circular wooden frames (Ø = 16 cm) and dried for 24 h in a laminar flow hood to achieve complete dehydration. During drying, the airflow rate was maintained at ~400 m³/h, with a constant temperature of 18°C. After drying, the samples were exposed to ultraviolet (UV) light for six hours to eliminate potential microbial contamination introduced during handling and processing. The dried hAM was then placed epithelial side up on cellulose nitrate filters, with orientation determined from the initial separation step (the upper layer corresponds to the epithelial side). AD-hAM was trimmed to sizes suitable for clinical use: 3 × 3 cm for ophthalmological applications and 8 × 8 cm for dermatological wounds and burns, based on standard requirements. The grafts were then packed in polyethylene envelopes, labeled, and heat-sealed. All procedures, including separation, washing, drying, and packaging, were performed under a Class II safety hood.

Hematoxylin and eosin staining for F-hAM and AD-hAM

**Figure 2:**
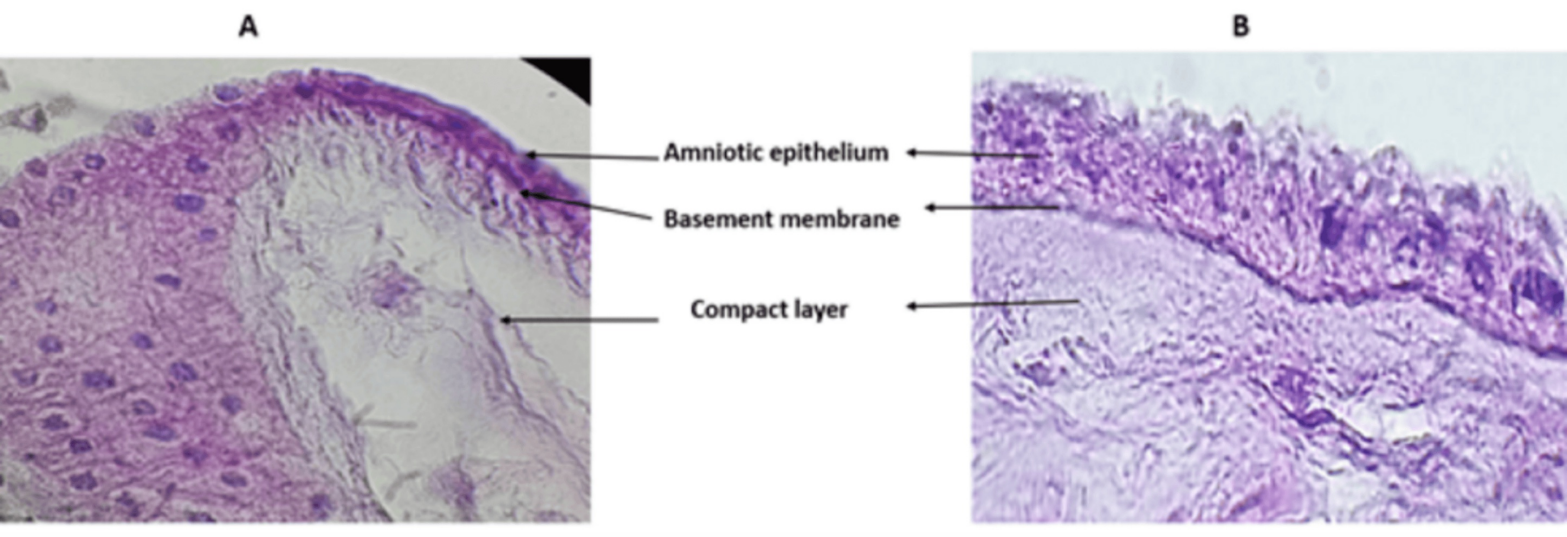
Histological micrographs of (A) F-hAM and (B) AD-hAM stained with hematoxylin and eosin (H&E). The stromal layer remained structurally intact in F-hAM. Partial epithelial detachment, decreased cell density, and a clear basement membrane can be seen in AD-hAM. F-hAM: frozen human amniotic membrane, AD-hAM: air-dried human amniotic membrane.

To assess structural changes before and after dehydration, representative samples were randomly selected from both F-hAM and AD-hAM. The fragments were fixed in 10% buffered formalin for 24 h at room temperature, sectioned at 4 µm, and processed using standard histological techniques. Sections were stained with hematoxylin and eosin and examined under a light microscope (Leica® DM-EP, Wetzlar, Germany). All images were captured at 40× magnification (Figure 2).

Determination of bioburden in AD-hAM before gamma irradiation

Bioburden (aerobic and anaerobic bacteria, yeast, and mold) was determined according to ISO 11737-1. One non-irradiated membrane package from each group (20-25 sachets produced from a single placenta) was randomly selected and opened after a minimum of 48 h for microbiological assessment. AD-hAM fragments were cut into small pieces and transferred to trypticase soya agar plates, then incubated at 30°C for 14 days. Thioglycolate broth was used for anaerobic bacteria at 37°C for 7 days under anaerobic conditions. Molds were cultured on Sabouraud dextrose agar and incubated at 30°C for 14 days. The detection limit of the culture method was ~1 CFU per plate.

Sterilization of AD-hAM

The vacuum-sealed AD-hAM was sterilized with gamma rays (Cobalt-60) at room temperature at the National Center for Nuclear Sciences and Technologies (Tunis). Samples were exposed to doses of 15, 20, and 25 kGy. The 25 kGy dose was included as it is the internationally accepted standard for tissue sterilization (ISO 11137), while lower doses (15 and 20 kGy) were tested to determine whether effective sterilization could be achieved with better preservation of structural integrity and biological activity.

The required Radiation Sterilization Dose (RSD) for a sterility assurance level (SAL) of $10^{-6}$ was calculated using the following equation: RSD = D10 (log bioburden - log SAL) kGy [30]. Sterilized AD-hAM packages were stored at 4°C until analysis, which was performed after one and two years to evaluate microbial permeability and bioburden.

Determination of bioburden and sterility after gamma irradiation of AD-hAM

Bioburden was determined by membrane filtration according to ISO 11737-1 (1995) using soybean casein digest agar. Sterility testing was performed in accordance with ISO 11137:2006. Two replicates from each batch of irradiated AD-hAM samples (n = 2 per group) were aseptically transferred to a saline blank containing 0.01% polysorbate and shaken for 30 minutes. The solution was then filtered aseptically through sterile 0.45 µm membrane filters. The filters were placed on soybean casein digest agar and incubated at 30°C for 14 days. The number of positive and negative sterility tests was recorded. A control test using the saline blank was included to exclude false-positive results.

Microbial permeability after storage

Sterile AD-hAM samples stored for one and two years were tested for impermeability against two Gram-positive (*Staphylococcus aureus*, *Streptococcus *sp.) and two Gram-negative (*Pseudomonas aeruginosa*, *Klebsiella pneumoniae*) bacteria. Both one-year and two-year samples were tested in the same experimental run to minimize inter-assay variability. Bacterial strains were obtained from the Microbiology Department. Fragments of hAM were placed on MacConkey agar and plate count agar plates. Bacterial suspensions were prepared in sterile water at a standardized concentration of 1 × 10⁶ CFU/mL, applied to the opposite surface of the membranes, and incubated for 24 h. Plates were then examined for microbial growth and for evidence of bacterial migration across the dressing surface.

Preparation of amniotic membrane extracts for total protein and growth factor analysis

For growth factor analysis, F-hAM samples were thawed, and 20 mg of each non-irradiated AD-hAM and AD-hAM irradiated at 15, 20, or 25 kGy were cut into small pieces, homogenized by vortexing in ice-cold PBS, and stored overnight at -20°C. After thawing, the homogenates were centrifuged at 5000 × g for 5 min at 2-8°C, and the supernatants were assayed immediately.

Comparison of total protein content of stored irradiated AD-hAM with frozen F-hAM

The total protein content of AD-hAM extracts was determined using the Bradford assay. Protein extracts were mixed with Bradford reagent (Bio-Rad, USA) and incubated at room temperature for 5-10 min. Absorbance was measured at 595 nm with a microplate reader (GENESYS 20, Thermo). Bovine serum albumin (BioLife, USA) was used as the standard. All measurements were performed in triplicate.

Enzyme-linked immunosorbent assay (ELISA)

Basic fibroblast growth factor (bFGF) and transforming growth factor-beta (TGF-β1) were quantified using ELISA kits (CUSABIO, USA) according to the manufacturer’s instructions. The detection ranges were 1.56-100 pg/mL for bFGF and 0.78-50 ng/mL for TGF-β1. Measurements were performed on F-hAM, as well as on non-irradiated and irradiated AD-hAM samples. Each cytokine was assayed twice in triplicate using an ELISA reader (BioTek, USA). Standard curves were generated for each assay, with R² values consistently above 0.98. Inter- and intra-assay variability were within acceptable limits.

Statistical analysis

Statistical analysis was performed using paired t-tests to compare total protein and growth factor levels between F-hAM and AD-hAM before and after irradiation at 15, 20, and 25 kGy. A p-value <0.05 was considered statistically significant.

## Results

Histological assessment of processed AD-hAM versus F-hAM

AD-hAM showed no visible morphological alterations following washing, air-drying, or UV exposure, and remained translucent and smooth. Hematoxylin and eosin (H&E) staining revealed that F-hAM preserved a continuous epithelial layer with a well-organized stromal structure, whereas AD-hAM exhibited partial epithelial detachment and reduced cell density, while the stromal layer remained structurally intact (Figure 2).

Bioburden in AD-hAM before gamma irradiation

No bacteria or mold was detected before final packaging and sterilization. After sterilization, no microbial growth was observed during 14 days of incubation at any radiation dose. Consequently, 25 kGy was adopted as the standard dose. Sterilized membranes were stored at 4°C until further analysis.

Bioburden in AD-hAM after one- and two-year storage

Sterilized AD-hAM samples stored at 4°C for one and two years were tested for microbial contamination. No bacterial or fungal growth was detected after 14 days of incubation in either group, confirming that gamma-irradiated AD-hAM remains sterile for at least two years under refrigerated storage.

Microbial barrier function

The microbial barrier function of irradiated AD-hAM stored at 4°C for one or two years was maintained. The membrane remained impermeable to both Gram-positive and Gram-negative bacteria (Table 1).

**Table 1:** Effects of storing irradiated air-dried amniotic membrane on its barrier properties.

	Impermeable after	
Bacteria	One year	Two years
Staphylococcus aureus	Yes	Yes
Streptococcus pyogenes	Yes	Yes
Pseudomonas aeruginosa	Yes	Yes
Klebsiella pneumoniae	Yes	Yes

Total protein content of F-hAM, non-AD-hAM, and irradiated AD-hAM

**Figure 3:**
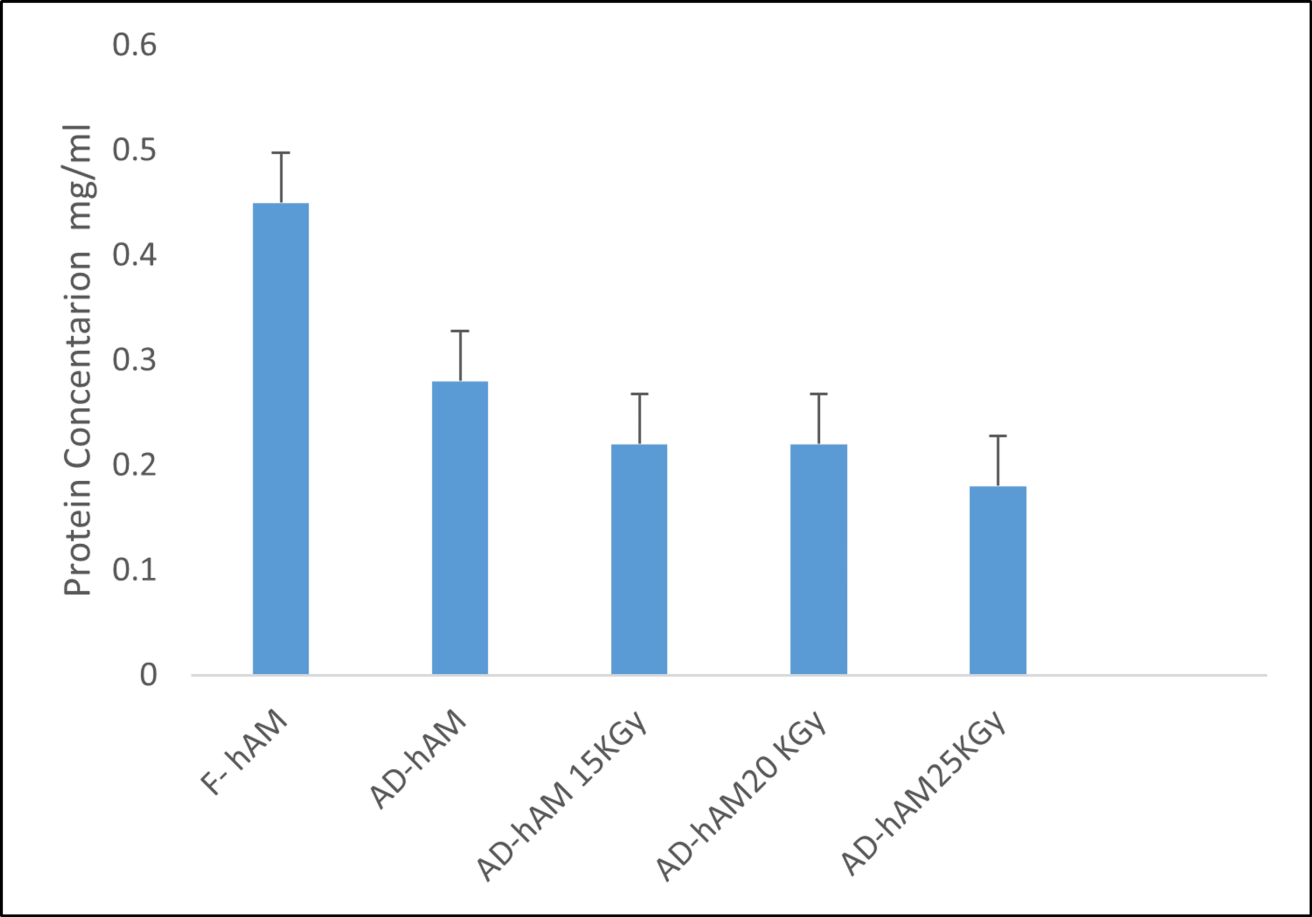
Total protein content of F-hAM, stored AD-hAM, and irradiated AD-hAM. F-hAM exhibited the highest protein concentration. A significant, dose-dependent reduction in protein content was observed in irradiated AD-hAM (p < 0.05). Error bars: standard deviation.

F-hAM showed the highest protein content (0.451 mg/mL) (Table 2, Figure 3). Irradiation caused a significant, dose-dependent reduction in protein levels in AD-hAM: 15 kGy, 0.268 mg/mL (p < 0.005); 20 kGy, 0.265 mg/mL (p < 0.006); and 25 kGy, 0.187 mg/mL (p < 0.003). AD-hAM stored for more than one year also demonstrated a significantly lower protein concentration (0.289 mg/mL) (p < 0.05).

**Table 2:** Total protein and growth factor levels in human amniotic membranes under different treatments. F-hAM: frozen human amniotic membrane, AD-hAM: air-dried human amniotic membrane, bFGF: basic fibroblast growth factor, TGF-β: transforming growth factor-beta.

Analyte	F-hAM	AD-hAM non-irradiated	p-value	AD-hAM (15 kGy)	p-value	AD-hAM (20 kGy)	p-value	AD-hAM (25 kGy)	p-value
Total protein, mg/mL	0.451	0.289	0.001	0.268	0.005	0.265	0.006	0.187	0.003
bFGF, pg/mL	79.5	63.4	0.002	56.1	0.001	54	0.0001	44.7	0.002
TGF-β, ng/mL	41.4	32.3	0.05	30	0.02	29	0.07	23	0.01

Levels of cytokines in irradiated AD-hAM compared with F-hAM after storage

**Figure 4:**
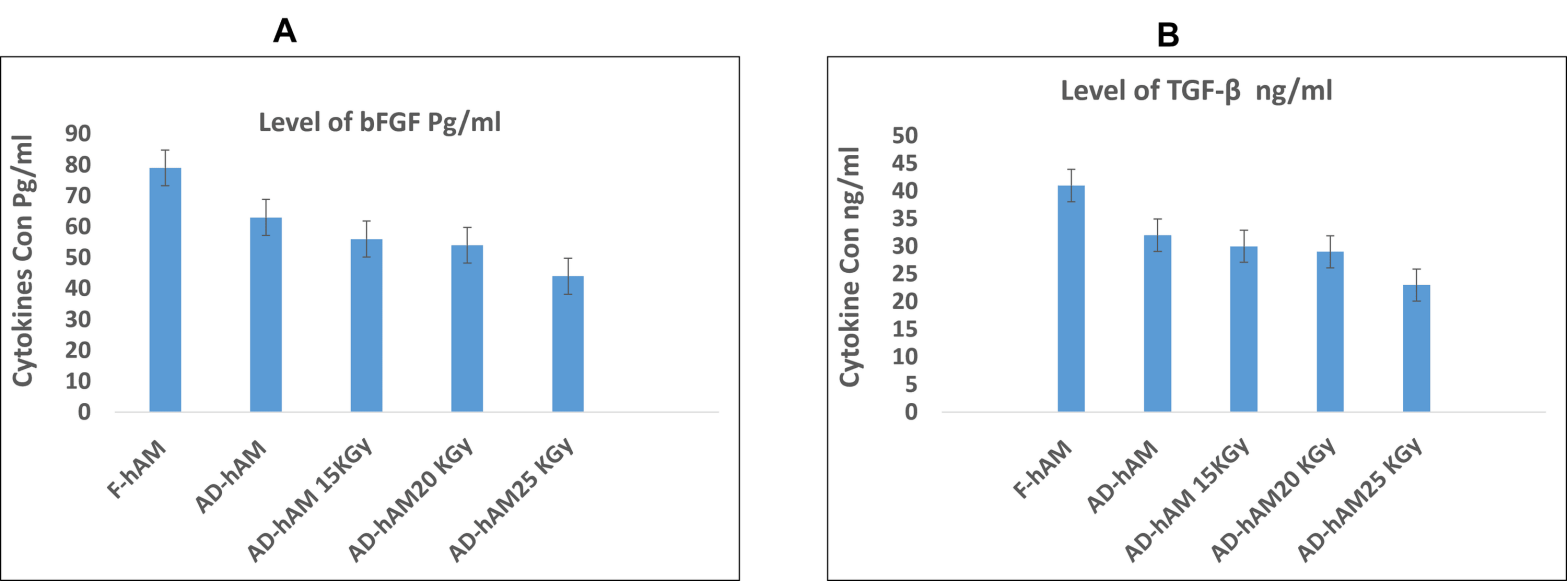
(A) Cytokine levels in F-hAM, stored non-irradiated AD-hAM, and stored irradiated AD-hAM. Concentrations of basic fibroblast growth factor (bFGF) and (B) transforming growth factor-beta 1 (TGF-β1) decreased significantly (p < 0.05) following storage and further declined with increasing doses of gamma irradiation. Error bars: standard deviation

Both bFGF and TGF-β1 levels (Figure 4) decreased after storage and with increasing irradiation doses. In F-hAM, bFGF concentration was 79.5 pg/mL, compared with 63.4 pg/mL in AD-hAM. After irradiation and storage for more than one year, bFGF levels declined further to 56.1 pg/mL (15 kGy), 54.0 pg/mL (20 kGy), and 44.7 pg/mL (25 kGy). Similarly, TGF-β1 levels decreased from 41.4 ng/mL in F-hAM to 32.3 ng/mL in AD-hAM and after irradiation dropped to 30.0 ng/mL (15 kGy), 29.0 ng/mL (20 kGy), and 23.0 ng/mL (25 kGy).

## Discussion

hAM have been widely utilized in tissue engineering and regenerative medicine due to their biocompatibility, antibacterial properties, immunomodulatory effects, and ability to support healing and reduce scarring. Fresh amniotic membrane is generally not preferred for wound dressings because of its limited shelf life and risk of transmitting infectious diseases, including HIV and other blood-borne infections [31]. To minimize this risk, tissue banks adopt measures such as careful donor screening, standardized processing, and sterilization, including gamma irradiation, which is the focus of this study [32]. Several methods are currently used to prepare and preserve hAM, such as air-drying, lyophilization (freeze-drying), and glycerol preservation [33]. Among these, air-drying is the most straightforward, requiring no specialized equipment, while producing grafts that can be stored at room temperature, making it a promising option for wound dressings [34]. In this study, we developed a simple, sterile AD-hAM with long-term storage capability. Histological evaluation showed that fresh hAM (F-hAM) retained a continuous epithelial layer and organized stromal matrix, whereas irradiated AD-hAM exhibited partial epithelial detachment and reduced cell density. These epithelial alterations may influence clinical performance, as epithelial integrity supports re-epithelialization and barrier protection during wound healing. However, the stromal architecture was largely preserved, suggesting that AD-hAM may still be useful in applications where stromal integrity is critical, such as scaffolding or wound coverage [35]. Interestingly, other studies have reported that “naked” AM (epithelium removed) can promote better adhesion, proliferation, and differentiation compared with intact AM [36]. Gamma-irradiated AD-hAM (15, 20, 25 kGy) preserved barrier function, preventing microbial infiltration even after two years of storage at 4 °C. These results are consistent with Bashandy et al. [37] and Singh et al., who observed intact barrier properties after up to five years of storage [38]. Sterility testing confirmed the absence of bacterial or fungal contamination before and after irradiation, in line with ISO 11737-1 standards. Air-drying and irradiation significantly reduced protein content from 0.451 mg/mL to 0.289 mg/mL (p < 0.05), consistent with prior reports that drying alters extracellular matrix components [39]. Air-drying causes greater protein and growth factor loss compared to lyophilization [40]. Although some studies suggest dehydration does not compromise growth factor activity [41], we found that both bFGF and TGF-β1 declined significantly after drying and irradiation in a dose-dependent manner. This is in agreement with Paolin et al., who reported cytokine reduction in irradiated hAM [42]. Higher irradiation doses have been associated with structural damage to all AM layers, yet several studies show that 25 kGy remains sufficient for sterilization while preserving functional activity. Djefal et al. demonstrated that irradiation at 25 kGy effectively sterilizes dehydrated hAM without major loss of bioactivity [43]. Thus, balancing microbial safety with preservation of biological function is essential. Lower doses (15-20 kGy) may better maintain growth factors and morphology, particularly in cryopreserved or lyophilized AM. Bioburden testing, conducted in accordance with ISO 11737-1 prior to terminal sterilization, confirmed that drying and handling did not introduce detectable bacteria or mold. After a 14-day incubation, all hAM samples remained free of microbial growth. To ensure reliability, strict cold-chain management and sterile conditions were maintained throughout processing. Sterility of AD-hAM was further validated through microbiological evaluation both before and after gamma irradiation. Our group has accumulated extensive experience in preparing air-dried and wet amniotic membranes [44] since 2006. This study has limitations. Donor variability may have influenced baseline cytokine levels and tissue quality. Only two growth factors (bFGF, TGF-β1) were assessed, limiting insight into the broader cytokine profile. The sample size was relatively small, which may reduce statistical power and generalizability. Finally, the absence of mechanical testing means structural and functional integrity could not be fully evaluated.

## Conclusions

This study presents a simple, sterile, and cost-effective protocol for preparing AD-hAM with long-term storage capability. The protocol ensures microbial safety while preserving stromal matrix integrity and barrier function after up to two years of storage. However, total protein content, including growth factors such as bFGF and TGF-β1, decreased in a dose-dependent manner, underscoring the need to balance sterilization with bioactivity preservation. Among the doses tested, 15 kGy appears most suitable, maintaining sterility with the least impact on growth factor levels and structural integrity.
